# Dementia / Alzheimer's Disease

**DOI:** 10.1186/1472-6874-4-S1-S20

**Published:** 2004-08-25

**Authors:** Joan Lindsay, Lori Anderson

**Affiliations:** 1 Department of Epidemiology and Community Medicine, University of Ottawa, 451 Smyth Road, Ottawa, Canada and Surveillance and Risk Assessment Division, Health Canada; 2 Private Consultant, Ottawa, Canada

## Abstract

**Health Issues:**

Dementia, including Alzheimer's disease (AD) increases exponentially with age from the age of 65. The number of people with dementia will increase significantly over the next three decades as the population ages. While prevalence and incidence rates do not differ markedly in women, compared to men, women live longer on average, so the *number *of women with dementia is greater than for men. Also, women are more frequently caregivers for people with dementia. Thus, dementia is an important health problem for women.

**Key Findings:**

The Canadian Study of Health and Aging showed an increase in prevalence of dementia with age for both sexes, approximately doubling every five years of age. Rates of AD were higher in women whereas rates of vascular dementia were higher in men. The risk of AD increased with increasing age, lower education, and apolipoprotein E ε4. Regular physical activity was clearly protective in women; this was less clear for men. Use of non-steroidal anti-inflammatory drugs, wine consumption, and past exposure to vaccines decreased the risk of AD. Estrogen replacement therapy did not reduce the risk of AD. About three quarters of caregivers for dementia patients were women.

**Data Gaps and Recommendations:**

The protective effect of regular physical activity for AD provides an additional reason to promote regular physical activity at all ages. Ongoing surveillance of the incidence, prevalence and risks for dementia is needed to monitor the impact of treatments as well as the aging of the population on the burden of dementia.

## Background

Dementia involves "a chronic deterioration of intellectual function and other cognitive skills severe enough to interfere with the ability to perform activities of daily living."[1] Dementia mainly affects seniors and also greatly affects their families and caregivers. It includes Alzheimer's disease (AD), the most common form of dementia, vascular dementia, and other, rarer, conditions. AD has been defined as "a progressive, inexorable loss of cognitive function associated with an excessive number of senile plaques in the cerebral cortex and subcortical gray matter, which also contains β-amyloid and neurofibrillary tangles consisting of tau protein."[1]

Two factors combine to make dementia an important issue for women: first, the Canadian population is aging, and the proportion of older people will increase rapidly over the next few decades as the baby boomers become seniors. The most rapid increase in the population is in the "oldest old," aged 85 and over, and this is the age group with the highest risk of dementia. Second, the average life expectancy is longer for women than men (81.1 years for women and 75.1 years for men),[2] which means that in older age groups the proportion of women increases. Thus more women than men develop and live with AD and other dementias.

With the aging of the Canadian population, it becomes increasingly important to understand the effects of dementia and AD on individuals, families, friends, caregivers, community health services and long-term care facilities. This review offers a brief summary of the magnitude of dementia in Canada, research on risk factors and preventive factors, caregiving issues, and health policy implications.

## Methods

### Data Source

The Canadian Study of Health and Aging (CSHA) is a longitudinal, multi-centre, population-based study that provides a great deal of detailed data on dementia in Canada. As the largest study of its kind, it is the only national, population-based study of dementia in the world. It was designed to focus on the prevalence, incidence, risk factors and caregiving patterns related to dementia in elderly Canadians. The study methods have been more thoroughly described elsewhere. [3-6]

During the first phase of the study (CSHA1), conducted in 1991–1992, representative samples of people aged 65 or older were randomly chosen from 36 urban and surrounding rural areas in the 10 Canadian provinces. The total sample was 10,263 people, including 9,008 community residents and 1,255 residents of institutions. There were 4,008 men and 6,255 women, providing a large sample that could be weighted to the population for population estimates of prevalence and incidence.

Community subjects were interviewed at home and screened for cognitive impairment using standardized, validated instruments. Those who were deemed to be impaired, a random sample of those who were not, and all participants in institutions were offered an extensive clinical evaluation involving a nurse, a physician, a psychometrist and a neuropsychologist. Consensus diagnoses classified subjects into one of the following categories: no cognitive impairment, cognitive impairment no dementia (CIND), AD (probable or possible), vascular dementia, other specific dementia, and unclassifiable dementia.

Subjects found to have AD for less than three years or to have vascular dementia became part of case-control studies of risk factors. [5-7] Control subjects were selected from those who screened negative and who were clinically diagnosed as cognitively normal. In addition, those deemed cognitively normal completed a questionnaire for prospective studies of risk factors. Risk factor data were obtained from proxies for both cases and controls for the initial retrospective analyses.

The caregiver study involved interviews with primary caregivers of subjects with dementia and caregivers of a sample group of subjects who were cognitively normal.

In the 1996–1997 study (CSHA2), the participants were followed up to measure changes in health status and functioning an average of five years after CSHA-1. Subjects went through the same diagnostic process as in CSHA-1. For participants who died before CSHA-2, the date and cause of death were obtained, and a relative or other informant was interviewed.

### Data Quality

The quality of the CSHA data is high. The initial community screening test was chosen in part because of its reliability and validity, and a high cut-point was used to increase sensitivity. Diagnosis of dementia, including subtypes, was made using carefully standardized, in-depth clinical examinations following a multi-stage protocol, and the latest criteria were applied. Response rates were high. All questionnaires were thoroughly checked and coded; data were double-entered until an error rate of less than 1 per 1,000 variables was reached.

## Results

### Prevalence

**Figure 1:**
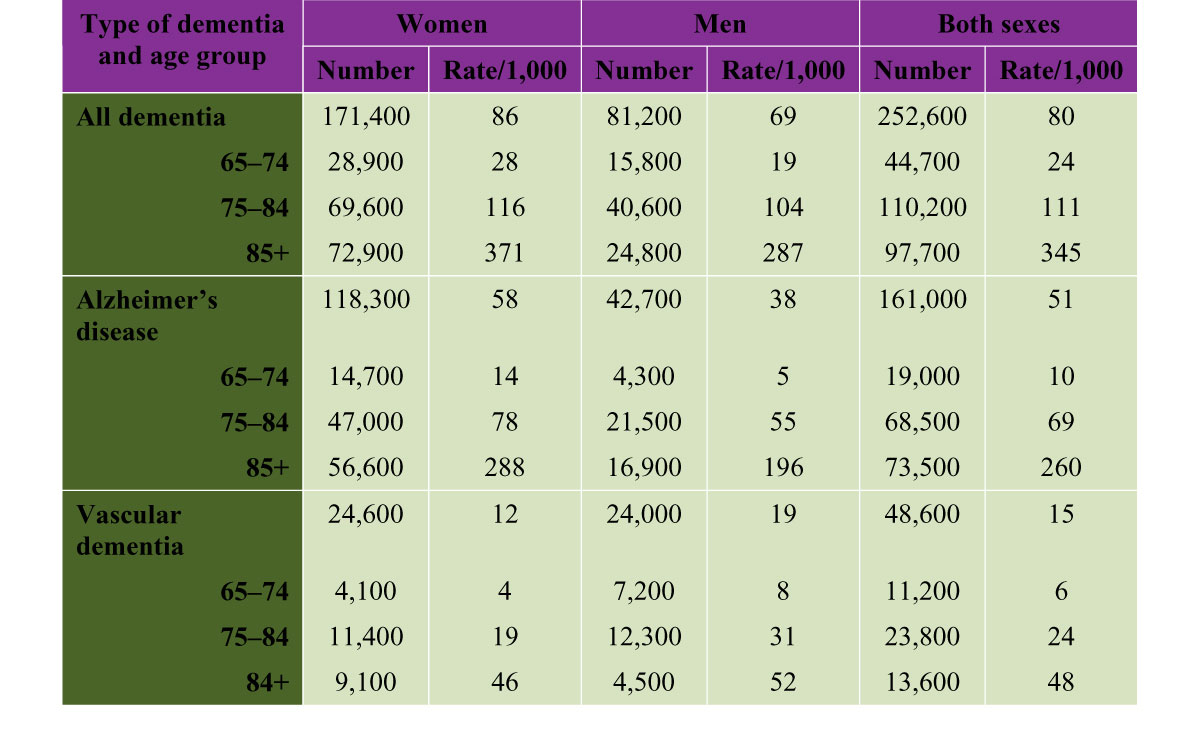
**Prevalence of dementia in Canada, 1991, by type of dementia, sex and age group – numbers and rates per 1,000 population **Source: Canadian Study of Health and Aging Working Group. Study methods and prevalence of dementia.[3]

From the CSHA-1 it was estimated that over a quarter of a million (252,600) seniors in Canada suffered from dementia in 1991, representing 8% of the population aged 65 or older (Figure 1).[3] More women than men had dementia (171,400 versus 81,200), although there was less difference in the prevalence rate (86 versus 69 per 1,000 population, respectively). This included 118,300 women and 42,700 men with AD, with rates of 58 and 38 per 1,000 population respectively.

Half (51%) of those with dementia were living in institutions. Of those with dementia living in the community, almost two thirds (63%) were women, whereas in institutions almost three quarters (72%) of those with dementia were women.

AD was the most common form of dementia in women; it was diagnosed in 69% of women with dementia versus 53% of men with dementia. In contrast, only 14% of women with dementia had vascular dementia, compared with 30% of men.

The rates of dementia increased markedly with age for both sexes. Among women, 28 per 1,000 of those aged 65 to 74 had dementia; this increased to 116 per 1,000 for women aged 75 to 84, and 371 per 1,000 for those aged 85 and over. The corresponding rates for men were 19, 104 and 287 per 1,000. Thus there was relatively little difference in the rates among men and women except in the 85+ age group. In contrast, the proportions of women in the two older age groups were substantially higher: 63% of those aged 75 to 84 with dementia were women, and 75% of those aged over 85.

**Figure 2:**
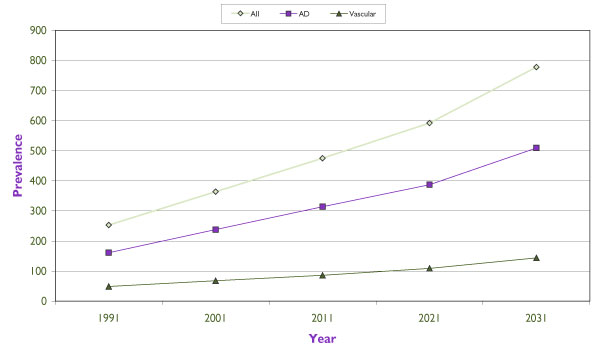
**Projected Prevalence of Dementia (× 1,000) Canada, 1991–2031. **Based on Table 11 in: Canadian Study of Health and Aging Working Group. Canadian Study of Health and Aging: study methods and prevalence of dementia. CMAJ 1994;150:899–913.

Projections of prevalent cases of dementia were calculated on the basis of the changing age composition of the Canadian population up to 2031 and are presented in Figure 2.[3] They are based entirely on the projected increases in the population aged 65 and over and assume no changes in prevalence due to changes in risk factors, treatment or survival.

### Incidence

Based on the 1991 population, the CSHA-2 follow-up study provided estimates of 60,150 incident (new) cases of dementia in Canada's population aged 65 and over each year.[6] The majority (60%) of new cases were women (36,320), and the overall age-standardized rate was 21.8 women per 1,000 non-demented population. The incidence rate among men was very similar (19.1 per 1,000 non-demented population). More than one fifth (22%) of new cases occurred in institutions. The approximate doubling of incidence rates with every five years of age has been seen in many studies.[8]

The CSHA prevalence and incidence rates of dementia seem to generally fall within the range of other community-based European and U.S. studies,[6,9-13] although towards the upper end of the distribution.[14] This may reflect the fact that the CSHA included both institutional and community samples as well as milder cases of dementia. The larger number of women with dementia, despite the comparatively small difference in prevalence and incidence rates, reflects the larger number of women in the population due to their longer life expectancy as well as their longer survival with dementia.

### Mortality

Dementia is a major risk factor for mortality. A follow-up investigation two years after the CSHA baseline study revealed that the greatest mortality risk was among women with dementia in institutions, whose risk of dying was six times as great as cognitively normal women in the community.[15] Five-year follow-up confirmed that elderly people with dementia had clearly increased mortality rates in all age-sex categories as compared with those who were cognitively normal.[16] Mortality increased strongly with age for people with all dementia subtypes.

Among women, those with vascular dementia experienced the worst prognosis, the five-year mortality ranging from 60% in the 65 to 74 age group to 83% in the over 85 group.[16] Among women with AD the prognosis was only slightly better, with a corresponding mortality range of 58% to 82%. Although the proportion of women dying within five years of CSHA-1 diagnosis was lower than among men in most of the dementia subgroups, the mortality rate ratios (mortality relative to the general population) were higher among women in most groups.

CSHA five-year follow-up data were also analyzed to estimate duration of survival from the onset of dementia symptoms, adjusting for length bias (failure to include people with rapidly progressive illness who died before being included in the study).[17] The unadjusted median survival among subjects with probable AD, possible AD and vascular dementia was 6.6 years, whereas the adjusted estimate was only 3.3 years. The median survival was 3.1 years for subjects with probable AD, 3.5 for possible AD and 3.3 for vascular dementia. Age at onset was the strongest predictor of survival: survival decreased as age at onset of dementia increased. Women survived for slightly longer with dementia than men (3.4 versus 3.2 years).

### Risk Factors for AD

The CSHA published data on risk factors for AD from a retrospective analysis of CSHA-1 data[5] and from a prospective analysis of incident cases.[18] We will focus on the longitudinal results, given the methodological advantage of this type of study design.

Briefly, the CSHA-1 analysis showed that age, family history of dementia, and lower educational level were significantly associated with an increased risk of AD.[5] Head injury slightly increased the risk, with borderline statistical significance. Study participants who had arthritis or who used non-steroidal anti-inflammatory drugs (NSAIDs) showed a significantly reduced risk of AD.

**Figure 3:**
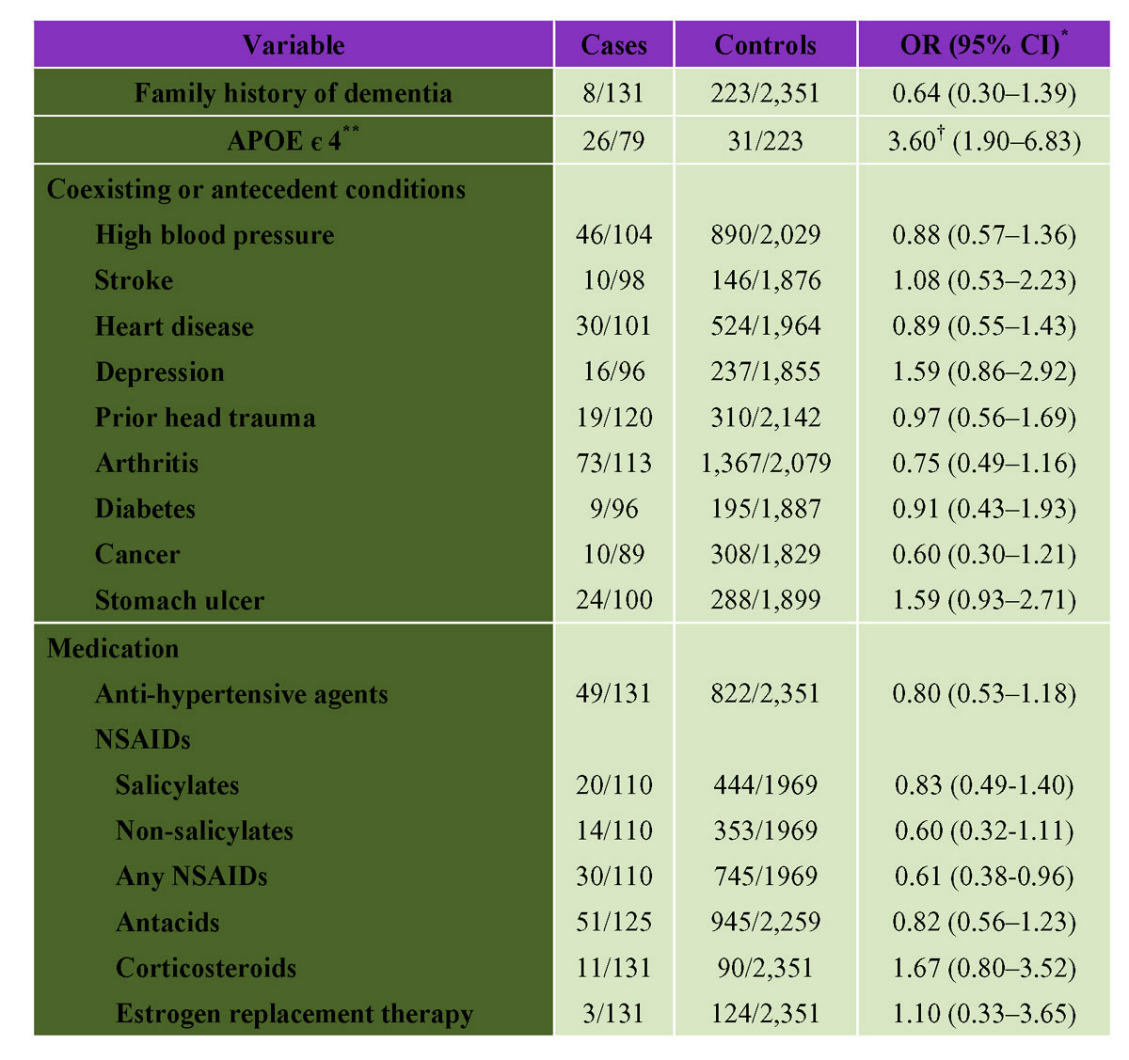
**Association of family history of dementia, APOE ε4 status, coexisting or antecedent selected diseases, and prior use of medication with risk of AD, **Canadian Study of Health and Aging, women, 1996. APOE = apolipoprotein E; NSAIDs = Non-steroidal anti-inflammatory drugs. OR = odds ratio; CI = confidence interval. * Adjusted for age and education. ** Comparison group is E3/E3. † Statistically significant

**Figure 4:**
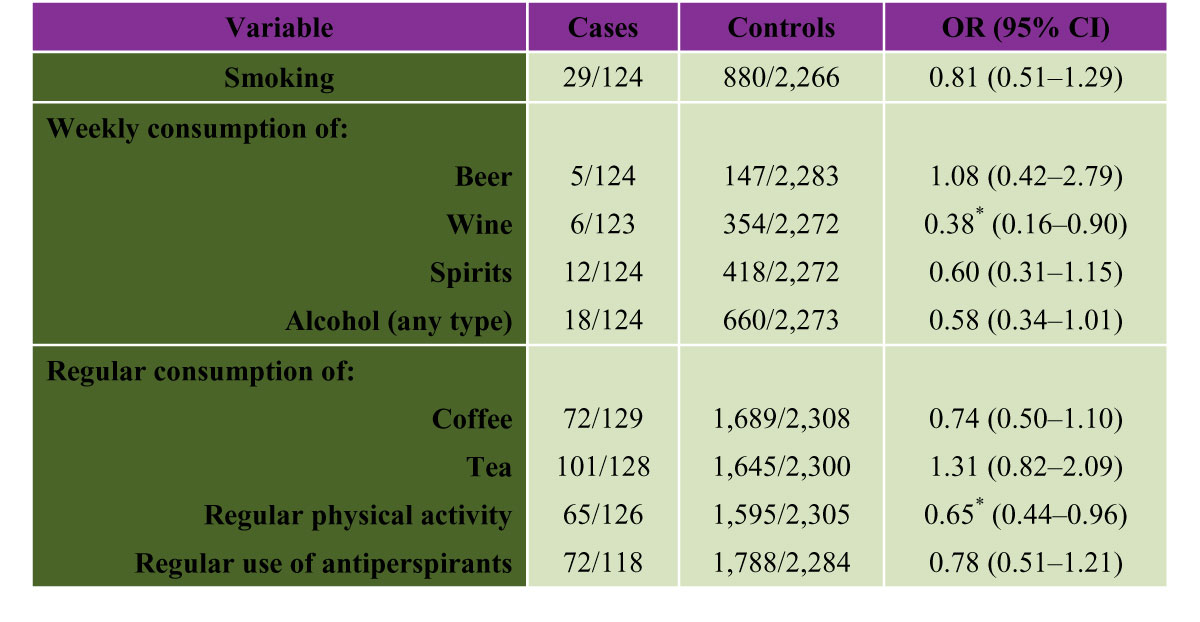
**Association of specific lifestyle variables with risk of AD, **Canadian Study of Health and Aging, women, 1996

Analysis of CSHA-2 prospective incidence data revealed a slightly different picture of risk factors for AD. Increasing age, low educational level and apolipoprotein E-ε4 (apoE-4) were significantly associated with increased risk of AD.[18] Regular physical activity, use of NSAIDs, wine consumption, past exposure to vaccines, and pre-existing arthritis were significantly associated with decreased risk of AD. [18-20] No statistically significant association was found for family history of dementia, sex, estrogen therapy, history of depression, head trauma, use of antiperspirant or antacid (containing aluminum), smoking, high blood pressure, heart disease or stroke (Figures 3 and 4).

Increasing age and the presence of the apoE-4 allele are solidly established risk factors for AD.[14,21] Although a positive family history of dementia is generally considered to be the only other definite risk factor, the CSHA-2 study did not find it to be significantly related to AD (contrary to the CSHA-1 prevalence study). However, these results are consistent with findings from the EURODEM pooled analysis of four European incidence studies.[22] It has been suggested that increased risk in first-degree relatives of AD patients may be related to relatively early onset of AD.[23]

The association of higher educational level with a reduced risk of AD is supported by several other prospective incidence studies in Europe and the United States.[22,24-27] Some of these studies found this association to exist for women only[27]or to be stronger for women.[26]

Notably, the CSHA-2 incidence study did not find sex to be a significant risk factor for AD.[18] A prospective study in East Boston also found that age-specific incidence of AD did not differ significantly by sex.[28] Prospective studies in Europe have identified female sex as a risk factor for incidence of AD, but only after age 85 or 90.[19,29]

Several protective factors for AD were identified in the CSHA-2 that have implications for preventive strategies. Arguably the most important was regular physical activity, a modifiable lifestyle habit that reduced the risk of AD by up to 50%.[20] Significant trends of increased protection with higher levels of physical activity were observed for AD and dementia of any type as well as for cognitive impairment with no dementia. The associations between physical activity and risk of CIND, dementia or AD were strongest in women, with a reduction in risk of AD of approximately 60% among women with high levels of physical activity, as compared with no physical activity. Although the literature is not entirely consistent, this finding has been supported by other studies.[18]

Regular use of NSAIDs was significantly related to a 35% reduction in the risk of AD in the CSHA-2 results.[18] This protective effect was observed in several population-based case-control studies, including the CSHA-1. [30-32] At least two other longitudinal studies have supported the CSHA-2 finding,[33,34] but others reported inconclusive results.[35,36] Although it is encouraging that use of NSAIDs may help to protect against AD, it also raises concern about potential side effects, particularly gastrointestinal bleeding.

Regular wine consumption was also significantly associated with reduced risk of AD (Odds Ratio [OR] 0.49) in CSHA-2 analyses,[18] and the protective effect appeared to be even stronger in women (OR 0.38). This protective relation is consistent with findings from a longitudinal study in France.[37] It has been suggested that specific substances in wine (not alcohol) have a positive effect on nerve cells.[38] Recently, however, a longitudinal study in the Netherlands found a significant association between light to moderate drinking and lower risk of dementia, but with no evidence of any variation by type of alcoholic beverage.[39] There are also the detrimental effects of alcohol to be considered.

Another possible protective factor for AD identified in CSHA-2 analyses was past exposure to vaccines against diphtheria or tetanus, poliomyelitis, and influenza.[19] After adjustment for age, sex and education, vaccines against diphtheria or tetanus and against poliomyelitis were significantly associated with lower risks of AD (60% and 40% lower respectively). Influenza vaccine was also related to a lower risk of AD, but the association did not reach statistical significance. Further adjustments for other lifestyle-related characteristics produced similar results. These findings are consistent with the hypothesis that changes in the immune system may be involved in aging and AD, but this remains to be verified in further epidemiologic and clinical studies.

Estrogen therapy in the past was thought to reduce the risk of AD. The CSHA did not see a protective effect,[18] and other studies have yielded inconsistent findings.[40,41] A longitudinal study in the United States found that estrogen use was not protective against cognitive decline related to aging, although the authors noted that their study included few women who used estrogen for two years or longer.[42] In contrast, a prospective study found that women who formerly used hormone therapy had a reduced risk of AD, as did current users whose treatment exceeded 10 years.[43] More recently, a redundant publication of the Women's Health Initiative has shown an increased risk of AD among women in the estrogen/progesterone arm of the study.[44]

### Informal Care

As dementia progresses in individuals, it affects not only them but also their caregivers. The CSHA-1 confirmed the presence of an extensive informal network of Canadians providing care to seniors with dementia.[4] Over 98% of the estimated 123,900 seniors with dementia in the community had a caregiver. Of these caregivers, 94% were informal caregivers, namely, unpaid family members or friends. Of the dementia patients in the community, 94% had two or more relatives or friends besides their primary caregiver who assisted them.

Women's strong caregiving role was clearly demonstrated in the CSHA data. Three quarters of the primary caregivers of those with dementia in the community were women, as were 71% of primary caregivers of dementia patients in institutions.[4] In the community, almost one quarter (24%) of these primary caregivers were wives, and 29% were daughters. Daughters represented 45% of the informal caregivers of institutionalized dementia patients.

Other characteristics of the informal primary caregivers of elderly dementia patients were notable. More than 70% were married, whether the senior lived in the community or an institution.[4] Friends and other family (i.e. neither spouses nor children) made up over one fifth of these caregivers in both residential settings. Caregivers in the community were less likely to be employed (29%) than caregivers of dementia patients in institutions (41%). The average age of community caregivers was 62, but 32% of them were aged 70 to 90;[45] 60% of the community caregivers lived with the dementia patient.

Caring for someone with dementia tends to negatively affect the well-being of the caregiver. Informal caregivers of seniors with dementia experienced more chronic health problems and depressive symptoms than those caring for a senior without dementia.[4] Caregivers of dementia patients in institutions were less likely to feel burdened and experienced less depression than the community caregivers, even though the severity of dementia was higher. After five years, caregivers of dementia patients who remained in the community reported significantly more burden than did caregivers of patients who moved into or remained in an institution.[46] A higher frequency of disturbing behaviours, community residence of the patient, and low informal support were associated with greater caregiver burden, which in turn led to more depression in the caregiver.[47] Furthermore, caregivers of dementia patients with more disturbing behaviours and greater functional disabilities received less help from family and friends than did caregivers of dementia patients in institutions.

Use of community care services in the CSHA-1 (in 1991) seemed low, although data were not collected on the availability of services.[4] Except for home physiotherapy, use of community services was higher among those caring for seniors with dementia than among those caring for seniors without dementia. However, when patients were matched for level of disability (measured by problems with activities of daily living), except for moderate disability, caregivers of dementia patients received *fewer *services than those of non-dementia patients.

Possible factors affecting access to community care services related to both care recipients and caregivers in the CSHA analyses. Having greater functional disabilities, living alone, living in a less urban environment and being older were identified as predictors of use of community care services by seniors with dementia.[48,49] Caregivers who were children of dementia patients used more services than those who were spouses.[4] In addition, caregivers who perceived their own health more poorly were more likely to use community services to help in caring for dementia patients.[48]

### Institutionalization

In 1991, it was estimated from CSHA-1 data that 51% of Canada's elderly population with dementia lived in institutions.[3] Five years later, CSHA-2 follow-up revealed that 51% of the dementia patients in the community-dwelling CSHA-1 caregiver study sample had been admitted to an institution.[50] The median time to admission was 41 months.

Similar to the use of community care services, the factors affecting the likelihood of admission to an institution involved characteristics of both dementia patients and their caregivers.[50] The patient's type of dementia, namely possible or probable AD, and the severity of disability in activities of daily living were significant predictors of admission to an institution. The caregiver's age over 60, not being a spouse or child of the patient, and severe caregiver burden were significantly associated with the care recipient's placement in an institution. Caregiver burden was strongly correlated with the patient's behavioural problems and the caregiver's depressive mood. The caregiver's desire to have the dementia patient admitted to an institution was related to the severity of the dementia, being unable to leave the patient alone, living with the patient, using two or more home care services, and level of caregiver burden.

In addition, the risk of being admitted to an institution was significantly higher among dementia patients living in Quebec, the Prairies or British Columbia than for those living in Ontario or the Atlantic provinces.[50]

Recent analyses of the CSHA data show that there was a significant relation between service use and the functional abilities (activities of daily living) of subjects, and that their physical limitations predicted use of services whereas cognitive problems did not.

## Discussion

### Limitations

The CSHA is internationally recognized as one of the best studies of the epidemiology of dementia; in addition it covered many other topics related to aging. It was well designed and conducted. More frequent follow-ups would have strengthened the study and reduced the uncertainties in calculating incidence that arose because of the larger number of people who died between phases of the study. Collection of risk factor data for the case-control analyses was limited by the abilities of proxies to respond for the study participants and provide the amount of detail that was requested. The same questions were asked of those who were cognitively normal, who completed risk factor information for the prospective analyses. This had the advantage of making the retrospective and prospective analyses comparable, but the disadvantage that there was not more detailed information for the prospective analyses. The caregiving data, with hindsight, might have benefited from the collection of additional information – for example, about the availability of services; however, this was not possible given time and resource constraints.

### Policy Implications and Recommendations

• Further study is needed to confirm some of the protective factors found in the CSHA and in other studies (coffee drinking, vaccinations, use of estrogen therapy) before policy recommendations can be made.

• The protective effect of regular physical activity for AD provides an additional reason for the promotion of regular physical activity at all ages. Education and ongoing mental activity are likely equally important.[51]

• Although the CSHA covers the years 1991 to 2001, ongoing surveillance of the incidence and prevalence of dementia is needed to monitor the impact of preventive strategies and treatments as well as the aging of the population. This is not feasible using the usual sources of data (hospitalizations, mortality, physicians' visits) since in only a fairly small fraction of dementia cases would dementia be listed as a diagnosis.

## Note

The views expressed in this report do not necessarily represent the views of the Canadian Population Health Initiative, the Canadian Institute for Health Information or Health Canada.
